# Nurse-based educational interventions in patients with peritoneal dialysis: A systematic review and meta-analysis

**DOI:** 10.1016/j.ijnsa.2022.100102

**Published:** 2022-09-24

**Authors:** Tanawin Nopsopon, Piyawat Kantagowit, Chitsanucha Chumsri, Piyaporn Towannang, Apinya Wechpradit, Nipa Aiyasanon, Ruchdaporn Phaichan, Talerngsak Kanjanabuch, Krit Pongpirul

**Affiliations:** a Department of Preventive and Social Medicine, Faculty of Medicine, Chulalongkorn University, Bangkok, Thailand; b Dialysis Policy & Practice Program (DiP3), School of Global Health, Faculty of Medicine, Chulalongkorn University, Bangkok, Thailand; c Division of Nephrology, Department of Medicine, King Chulalongkorn Memorial Hospital, Bangkok, Thailand; d Dialysis Unit, Udonthani Hospital, Udon Thani, Thailand; e Medical and Psychiatric Nursing Division, Department of Nursing, Faculty of Medicine, Siriraj Hospital, Mahidol University, Bangkok, Thailand; f Respiratory Intensive Critical Care Unit, Chaophraya Abhaibhubejhr Hospital, Prachin Buri, Thailand; g Center of Excellence in Kidney Metabolic Disorders and Division of Nephrology, Department of Medicine, Faculty of Medicine, Chulalongkorn University, Bangkok, Thailand; h Peritoneal Dialysis Excellent Center, King Chulalongkorn Memorial Hospital, Bangkok, Thailand; i Department of International Health, Johns Hopkins Bloomberg School of Public Health, Baltimore, MD, USA; j Bumrungrad International Hospital, Bangkok, Thailand

**Keywords:** Education, Meta-analysis, Nurses, Nursing intervention, Nephrology nursing, Peritonitis, Peritoneal dialysis, Review

## Abstract

**Background:**

Peritoneal dialysis (PD) is a major renal replacement therapy modality for patients with end-stage kidney disease (ESKD) worldwide. As poor self-care of PD patients could lead to serious complications, including peritonitis, exit-site infection, technique failure, and death; several nurse-based educational interventions have been introduced. However, these interventions varied and have been supported by small-scale studies so the effectiveness of nurse-based educational interventions on clinical outcomes of PD patients has been inconclusive.

**Objectives:**

To evaluate the effectiveness of nurse-based education interventions in PD patients.

**Design:**

A systematic review and meta-analysis of Randomized Controlled Trials (RCTs).

**Methods:**

We performed a systematic search using PubMed, Embase, and CENTRAL up to December 31, 2021. Selection criteria included Randomized Controlled Trials (RCTs) relevant to nurse-based education interventions in ESKD patients with PD in the English language. The meta-analyses were conducted using a random-effects model to evaluate the summary outcomes of peritonitis, PD-related infection, mortality, transfer to hemodialysis, and quality of life (QoL).

**Results:**

From 9,816 potential studies, 71 theme-related abstracts were selected for further full-text articles screening against eligibility criteria. As a result, eleven studies (1,506 PD patients in seven countries) were included in our systematic review. Of eleven studies, eight studies (1,363 PD patients in five countries) were included in the meta-analysis. Sleep QoL in the intervention group was statistically significantly higher than control (mean difference = 12.76, 95% confidence intervals 5.26–20.27). There was no difference between intervention and control groups on peritonitis, PD-related infection, HD transfer, and overall QoL.

**Conclusions:**

Nurse-based educational interventions could help reduce some PD complications, of which only the sleep QoL showed statistically significant improvement. High-quality evidence on the nurse-based educational interventions was limited and more RCTs are needed to provide more robust outcomes.

**Tweetable abstract:**

Nurse-based educational interventions showed promising sleep quality improvement and potential peritonitis risk reduction among PD patients.

**What is already known**

• Nephrology nurses play an important specialized education role, especially in patients with peritoneal dialysis (PD).
• Several nurse-based educational interventions have been introduced to reduce the complications of PD and improve quality of life (QoL).
• The effects of nurse-based educational interventions on clinical outcomes including peritonitis, exit-site infections, technique failure, mortality, and QoL of patients with peritoneal dialysis have remained inconclusive.

**What this paper adds**

• Nurse-based educational interventions could improve sleep quality and may potentially reduce risk of peritonitis in PD patients.
• More efficacious education and contact time of nurses given to PD patients could possibly contribute to more protection against peritonitis, exit-site infections, and PD-related infections.

## 1 Introduction

Peritoneal dialysis (PD) is a cost-effective dialysis modality for patients with end-stage kidney disease (ESKD) worldwide. In the literature survey across 46 countries, 94% of published studies demonstrated that PD is less costly than hemodialysis (HD), with PD costing at least 25% less in 22 countries (Karopadi et al., 2013). Recent nationwide studies also showed the same trend of cost ratio between PD and HD (Ferguson et al., 2021, Chang et al., 2016). PD is associated with a similar long-term survival (Wong et al., 2018), albeit offering benefits in short-term outcomes compared to HD (Li and Chow, 2013). Moreover, patients receiving PD experience superior treatment satisfaction and longer preservation of residual kidney function (Rubin et al., 2004, Moist et al., 2000, Misra et al., 2001, Mathew et al., 2016). Thus, the use of PD is increasing in many countries, including the US, China, and Thailand (Li et al., 2017). However, efforts to increase PD utilization are limited in the other countries by concerning peritonitis and the shortened treatment time on PD compared to HD. The Standardized Outcomes in Nephrology-Peritoneal Dialysis study established peritonitis and PD technique failure as core outcomes of importance to clinicians, patients, and stakeholders (Manera et al., 2020). To prevent peritonitis, patients and/or caregivers require nurse-based education and training to perform high-quality PD, as they perform PD in their homes without direct assistance or supervision from healthcare providers. Poor adherence to the PD exchange procedure is associated with an increased risk of PD peritonitis (Mawar et al., 2012, Dong and Chen, 2010). Several nurse-based educational interventions in randomized controlled trial (RCT) fashion have been introduced to mitigate the PD-related complications. For example, Chang et al., 2018 demonstrated that frequent patient retraining at home by PD nurses reduced the risk of the first episode of peritonitis compared to conventional training protocol in 104 patients (Chang et al., 2018). Xu et al., 2020 highlighted that nurse-based educational interventions reduced the time to first peritonitis of PD patients compared to usual care in 150 incident Chinese patients (Xu et al., 2020).

Although several RCTs have been conducted to provide solid evidence and highlight the strength of nurse-based educational interventions on ESKD patients receiving PD, all of the studies had small sample sizes, causing limited generalizability of the findings. Hence, we conducted a systematic review to evaluate the effectiveness of nurse-based educational interventions.

## 2 Methods

This study was conducted following the recommendations of the Preferred Reporting Items for Systematic Review and Meta-Analysis Protocols (PRISMA-P) 2015 statement (Shamseer et al., 2015). PRISMA-P 2015 checklist was provided in **Supplementary Material 1**. We prospectively registered the systematic review with PROSPERO International Prospective Register of Ongoing Systematic Reviews (Registration number: CRD42021250731).

### 2.1 Search strategy

We worked with an information specialist to identify original peer-reviewed RCTs to design a proper search strategy. PubMed, Embase, and the Cochrane Central Register of Controlled Trials (CENTRAL) were used to systematically search for RCTs up to December 31, 2021. The terms “peritoneal dialysis” and “patient education” were used in combination with “randomized controlled trial” as the keywords for literature search along with their synonyms. The entire search strategy is presented in **Supplementary Material 2**. In addition, the reference lists of included articles were searched, and related citations from other journals via Google Scholar. The electronic search was not limited to any beginning date to minimize the biases and achieve the validity of the search.

### 2.2 Study selection

Article selection was done by two independent reviewers for eligible studies, according to the following inclusion criteria: (i) original articles of RCTs of interventions, (ii) patients with nurse-based educational interventions or standard treatment of PD in combination with nurse-based educational interventions, (iii) at least one of the following outcomes specified in original articles: peritonitis, PD-related infection, technique failure or HD transfer, QoL, or death, (iv) English language, and (v) peer-reviewed articles. Exclusion criteria were: (i) duplicate reports or analysis failing to report additional outcomes, (ii) non-random treatment allocation, (iii) non-peer-reviewed articles, and (iv) studies on patients with ESKD without PD modality. Discrepancies between the two reviewers were resolved by consensus and another reviewer.

### 2.3 Outcomes of interest

The primary outcomes were the peritonitis rates, exit site infection event rates, PD-related infection event rates, and QoL. Peritonitis rates were defined as event rates of peritonitis per patient-year of intervention groups compared to control groups after randomization. PD-related infection event rates were defined as event rates of infection related to PD per patient-year of intervention groups compared to control groups. Secondary outcomes were technique failure or HD transfer event rates, mortality rates, and adverse events. We gathered data for time periods. We collected data from each research and determined the longest durations of each data.

### 2.4 Data extraction

Data extraction was done by two independent reviewers for published summary estimate data. Discrepancies between the two reviewers were resolved by consensus and another reviewer. We extracted the following data: (i) study information (authors, year of publication, study type, journal, contact, country, and funding), (ii) characteristics of the participants (sample size, age, gender, disease duration), (iii) intervention detail (type of intervention, duration of treatment, dosage, route and location of administration); (iv) comparator detail (type of comparator, duration of treatment, dosage, route and location of administration); and (v) outcomes (complete list of the names of all measured outcomes, unit of measurement, follow-up time point, location of measurement, measurement device, missing data). All relevant text, tables, and figures were examined for data extraction. We did contact the RCT authors to request incompletely reported data. If the RCT authors did not respond for 14 days, we conducted analyses using available data.

### 2.5 Quality assessment

Two independent authors assessed the risk of bias in the included RCTs using the Cochrane Risk of Bias tool 2.0 for RCT study (Sterne et al., 2019). We assessed the randomization process, deviations from intended intervention, missing outcome data, measurement of the outcome, and selection of the reported result. We assigned each domain as low risk of bias, unclear risk of bias, and high risk of bias. We did contact the RCT authors if there is insufficient information to assess. If the trial authors did not respond for 14 days, we assessed the available data. The disagreement between the two authors was resolved through discussion and another reviewer.

### 2.6 Data synthesis & statistical analysis

The term nurse-based educational intervention was defined as any educational interventions including telephoning, visiting, procedure, monitoring, and teaching program in which nurses participate in the interventions. Meta-analyses were conducted to compare the outcome of interests of intervention groups to control groups by using the relative risk (RR) method for dichotomous outcomes and the mean difference (MD) method for continuous outcomes. The outcomes conducted in the meta-analysis were the longest follow-up time of the outcomes in the study. Meta-analysis was conducted with 95% confidence intervals (CI) and p-value (p). p < 0.05 was considered statistically significant.

We assessed clinical and methodological heterogeneity by examining participants’ characteristics, follow-up period, outcomes, and comparators. We then assessed statistical heterogeneity using the *I^2^* statistic for magnitude, direction, and strength of evidence for heterogeneity. We regarded level of heterogeneity for I² statistic as defined in chapter 9 of the Cochrane Handbook for Systematic Reviews of Interventions: 0% to 40% might not be important; 30% to 60% may represent moderate heterogeneity; 50% to 90% may represent substantial heterogeneity; 75% to 100% considerable heterogeneity (Higgins et al., 2019). The random-effects meta-analysis by DerSimonian and Laird method—a variation of the inverse-variance method—was used for clinical, methodological, and statistical heterogeneity. Prespecified subgroup analysis for the type of intervention was performed. Additionally, sensitivity analyses were considered repeating the meta-analysis to determine the statistical robustness of the primary outcome by removing one study at a time. Because fewer than 10 studies were included in a meta-analysis, meta-regression was not done for further analysis. We also did not assess publication bias from a funnel plot due to fewer than 10 included studies in the meta-analysis (Sterne et al., 2011). The significant asymmetry indicated the possibility of publication bias or heterogeneity. The meta-analysis was performed using Review Manager version 5.3 (Review Manager 2014).

## 3 Results

### 3.1 Study selection

**Fig. 1:**
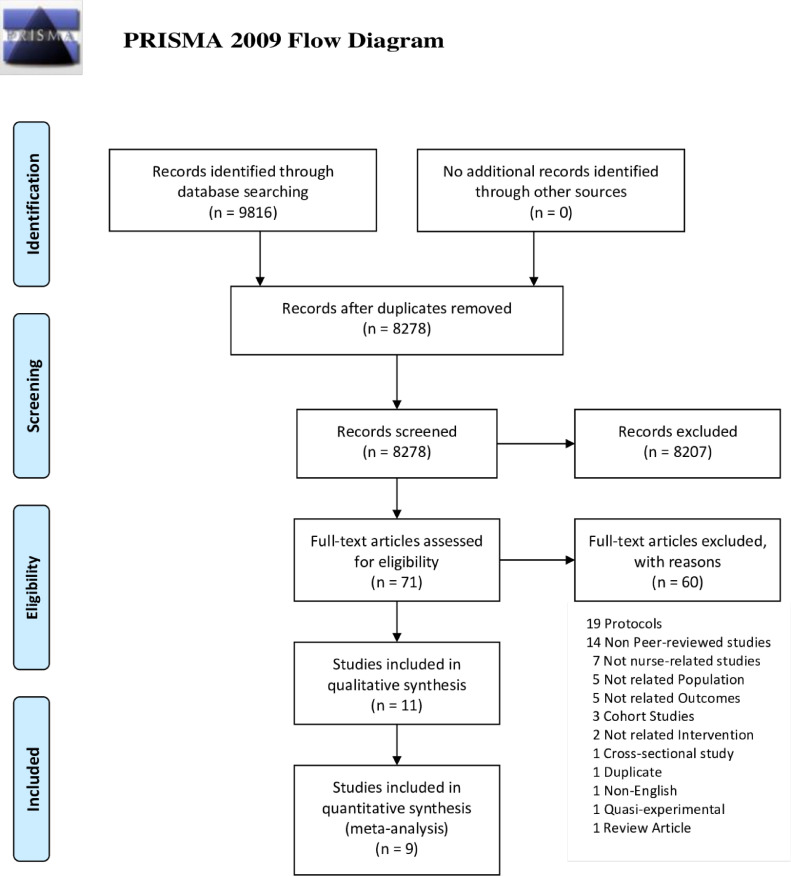
Flow chart diagram presenting the study selection with Preferred Reporting Items for Systematic Reviews and Meta-analyses (PRISMA) guidelines.

The systematic database search identified 9,816 potential articles. After duplicates removal, 8,278 titles passed the title and abstract screening, and 71 relevant abstracts were selected for full-text articles screening against eligibility criteria (Fig. 1). A total of 60 records were excluded for the following reasons: 19 protocols, 14 non-peer-reviewed studies, seven not nurse-related studies, five not PD populations, five not related outcomes, three cohort studies, two not nurse-related interventions, one cross-sectional study, one duplicate, one not English, one quasi-experimental study, and one review article. Eleven studies (Chang et al., 2018, Xu et al., 2020, Chow and Wong, 2010, Hare et al., 2014, Ljungman et al., 2020, Luo et al., 2019, Luo et al., 2020, Wong et al., 2010, Pungchompoo et al., 2020, Chen et al., 2008, Li et al., 2021) were included in the systematic review, and only eight (Chang et al., 2018, Xu et al., 2020, Chow and Wong, 2010, Ljungman et al., 2020, Luo et al., 2019, Luo et al., 2020, Wong et al., 2010, Pungchompoo et al., 2020) were included in the meta-analysis.

### 3.2 Characteristics of the studies

The eleven included RCTs were published between 2008 and 2021 from seven countries in three World Health Organization (WHO) regions. The number of patients per study ranged from 15 to 671, with a total of 1,506 PD patients, of which 588 (39.0%) were females. The mean age of studies varied from 42.1 to 62.4 years. The mean baseline duration of dialysis from 4 studies (Chow and Wong, 2010, Hare et al., 2014, Wong et al., 2010, Chen et al., 2008) ranged from 18.2 to 42.0 months. Causes of ESKD, including chronic glomerular disease, hypertension, diabetes mellitus, systemic lupus erythematosus, was shown in six studies (Chang et al., 2018, Chow and Wong, 2010, Ljungman et al., 2020, Luo et al., 2019, Luo et al., 2020, Chen et al., 2008) and were different in detail across the studies. Nine studies (Chang et al., 2018, Xu et al., 2020, Chow and Wong, 2010, Hare et al., 2014, Luo et al., 2019, Luo et al., 2020, Wong et al., 2010, Pungchompoo et al., 2020, Li et al., 2021) demonstrated educational levels of participants using numerous scales depending on the conducting country of the study. Six studies (Chow and Wong, 2010, Hare et al., 2014, Luo et al., 2019, Luo et al., 2020, Wong et al., 2010, Pungchompoo et al., 2020) reported marital status. The full characteristics of the studies including intervention type, control type, and duration of follow-up are demonstrated in Table 1. The detail of the intervention and control group are provided in Table 2.

**Table 1:** Characteristics of included PD studies and populations.

**Study**	**Countries**	**Income group**	**WHO Regions**	**Total N**	**Female n (%)**	**Mean age (SD)**	**Kidney function baseline**	**Duration of dialysis mean (SD), mo**	**PD modality(CAPD %)**	**Cause of ESKD**	**Education levels**	**Marital status (Single, Married, Divorced)**	**Comparison**		**Follow- up time(mo)**
													**Interventiongroup**	**Controlgroup**	
Chang 2018 (Chang et al., 2018)	South Korea	HICs	Western Pacific	104	37 (36%)	50.0 (11.9)	RRF 6.4 (5.02) mL/min/ 1.73m^2^	N/A	95.2	CGN 20.2%, HT 11.5%, DM 54.8%, Others 13.5%	≤9 yr: 24.0%, 9-12 yr: 31.7%, ≥12 yr: 44.23%	N/A	Frequent retraining (Education) and monitor	Conventional training	24
Chen 2008 (Chen et al., 2008)	Taiwan	HICs	Western Pacific	26	11 (42%)	50.3 (11.9)	RRF 0.14 (0.28) mL/min/ 1.73m^2^	40.8 (29.2)	70.8	CGN 45.8%, HT 8.3%, DM 20.8%, Others 25.0%	N/A	N/A	Sleep hygiene education with cognitive behavioral therapy (CBT)	Sleep hygiene education	1
Chow 2010 (Chow and Wong, 2010)	Hong Kong	HICs	Western Pacific	85	33 (39%)	56.9 (13.5)	N/A	38.4 (31.2)	N/A	*CGN 1.2%, HT 10.6%, DM 24.7%, SLE 1.2%, Others 63.5%	no formal study 10.6%, primary 41.2%, secondary 40.0%, above secondary 8.2%	18.8%, 71.8%, 9.4%	Comprehensive education program and follow up	Routine discharge services	3
Li 2021 (Li et al., 2021)	China	UMICs	Western Pacific	102	48 (47%)	42.1 (9.48)	N/A	N/A	48 %	N/A	elementary school 20.5%, Junior high school 30.0%, High school and above 49.5%,	N/A	CLHM system based on an internet platform (education, monitoring)	Routine care	12
Hare 2014 (Hare et al., 2014)	UK	HICs	European	15	1 (7%)	60.1 (11.2)	N/A	18.2 (14.3)	N/A	N/A	none (26.7), school (6.7), diploma (13.3), vocation (26.7), university degree (6.7), postgraduate (13.3)	80.0%, 13.3%, 6.7%	LIP (Education)	Deferred entry of LIP	5
Ljungman 2020 (Ljungman et al., 2020)	Sweden	HICs	European	671	225 (34%)	60.2 (14.5)	Serum Cr 7.3 (2.5)	N/A	80.5	CGN 25.9%, DM 22.4%, Others 51.7%	N/A	N/A	New follow-up model (Follow-up, monitor, education)	Standard care	13.7 for IG, 17.0 for CG
Luo 2019 (Luo et al., 2019)	China	UMICs	Western Pacific	128	58 (45%)	54.3 (12.7)	RRF 2.71 (0.69) mL/min/ 1.73m^2^	3-12 mo: 25.0%, 12-36 mo: 50.8%, >36 mo: 24.2% mean: N/A	100	CGN 33.6%, HT 25.8%, DM 23.4%, SLE 10.2%, Others 7.0%	Junior high school or below 40.6%, High school 34.4%, College or above 25.0%	18.0%, 60.2%, 21.8%	Health Education	Routine care and follow-up	12
Luo 2020 (Luo et al., 2020)	China	UMICs	Western Pacific	135	61 (45%)	55.6 (14.8)	N/A	3-12 mo: 24.5%, 12-36 mo: 52.6%, >36 mo: 22.9% mean: N/A	100	CGN 34.1%, HT 24.4%, DM 22.9%, SLE 10.4%, Others 8.2%	Junior high school or below 39.3%, High school 35.6%, College or above 25.2%	17.8%, 68.1%, 14.1%	Food exchange model intervention (Counseling, Follow-up, Monitor)	Routine dietary guidance and care	12
Pungchompoo 2019 (Pungchompoo et al., 2020)	Thailand	UMICs	South-East Asian	41	22 (54%)	N/A	Residual urine, mean; 403.91	<12 mo: 19.5, 12-36 mo: 7.3, >36 mo: 48.8% mean: N/A	100	N/A	no education 12.2%, primary 48.8%, secondary 22.0%, above (diploma, bachelor) 39.0%	12.20%, 73.17%, 14.63%	Self-Management Retraining Program (Education, monitoring, consulting)	Usual standard care	6
Wong 2010 (Wong et al., 2010)	Hong Kong	HICs	Western Pacific	49	23 (47%)	62.4	N/A	42.0	100	N/A	no formal study 20.4%, primary 41.8%, secondary 33.7%	N/A, 63.3%, N/A	Disease Management Program (Monitoring/Follow-up/Counseling)	Routine care	3.25
Xu 2019 (Xu et al., 2020)	China	UMICs	Western Pacific	150	69 (46%)	54.6 (14.8)	N/A	N/A	90.0	N/A	high school or above 54%	N/A	I1 = Technique inspection (Monitor), I2 = Oral education,	Usual care	48.4 ± 20.6

**Abbreviations**: CG, control group; CGN, chronic glomerular disease; Cr, creatinine; DM, diabetes mellitus; ESKD, end-stage kidney diseases; HICs, high income countries; HT, hypertension; IG, intervention group; LIP, Liquid Intake Program; mo, month(s); N/A, not available; PD, peritoneal dialysis; RRF, residual renal function; SD, standard deviation; SLE, systemic lupus erythematosus; UMICs, Upper middle-income countries; yr; year(s).

* More than 100% of the Information collected from the article

**Table 2:** Details of interventions in included trials.

**Study**	**Details of interventions**	
	**Intervention group**	**Control group**
Chang 2018 (Chang et al., 2018)	Frequent retraining program: over the trial period, subjects in the frequent retraining group received more frequent training visits (education) and monitoring than the conventional training group. In addition to two conventional sessions, the subjects received extra home visits for regular retraining at months 4, 5, 6, 7, 8, 10, 12, 15, 18, 21, and 24 by the PD nurse. The home training sessions were an hour in length. The content and curriculum of the home training visit were the same for both groups and based on ISPD guidelines, including an overview of PD, aseptic technique, hand washing, exchange procedure, exit site care, diet, and management of complications.	Conventional training: participants were given two sessions of training at week 1 and month 2 in their homes by PD nurses.
Chen 2008 (Chen et al., 2008)	Sleep hygiene education with CBT: after receiving the initial sleep hygiene session, participants were delivered four one-hour-weekly sessions of CBT during the 4-week period. A nurse specialized in PD, attended, and closely monitored the intervention.	Sleep hygiene education: receiving one sleep hygiene session at the start of the trial.
Chow 2010 (Chow and Wong, 2010)	Comprehensive education planning protocol and nurse-initiated telephone weekly follow-up for 6 weeks: comprehensive education planning was comprised of the individualized education program and conducted by the nurse care manager.	Routine discharge services
Li 2021 (Li et al., 2021)	CLHM system based on an internet platform: the health management system was accessible by the patient or his family members via a mobile phone for in-hospital and out-of-hospital management. The health management plans were considered and produced by the team members and organized relevant knowledge training for intervention personnel regularly. PD specialist nurses formulated personalized nursing plans to help strengthen confidence to manage the disease and were responsible for educational activities, including personal guidance, distribution of health brochures, group lectures, etc..	Routine care, including medications, health education, psychological care etc., guidance in the PD method 1 day before discharge, and advised patients to follow up on time.
Hare 2014 (Hare et al., 2014)	LIP (Education): LIP was delivered in a group of six to eight people format for a one-hour session, once a week for 4 weeks, in a hospital education room. Fluid non-adherence identified by fluid overload using standard clinical assessment and clinical judgment made by the PD medical team (PD nurses and consultant nephrologists)	Deferred entry of LIP
Ljungman 2020 (Ljungman et al., 2020)	Retraining group underwent testing including practical tests and questionnaires at all follow-up visits at months 1, 3, 6, 12, 18, 24, 30, 36. The testing required 2 to 2.5 hours and took place at either the PD center or in the patients’ homes.	Standard care and follow-up based on ISPD.
Luo 2019 (Luo et al., 2019)	Health Education by nursing-led MDT comprising of 2 kidney attending physicians, 2 PD specialized nurses, a clinical dietitian, a clinical psychotherapist, a physical therapist, some social workers, and a postgraduate volunteer. Nurse-led MDT care and follow-up based on 5E's renal rehabilitation program (encouragement, education, exercise, employment, and evaluation).	Routine care and follow-up such as telephone follow-up, outpatient follow-up, and routine health education conducted by a specialized nurse.
Luo 2020 (Luo et al., 2020)	Nurse-led food exchange model intervention: the patients were received a 24-hour dietary review form by nurses during their follow-up visit in the PD center to record their daily food and liquid intake at home. After reviewing the record, personalized diet plans were created. During the home visit, telephone follow-up, and outpatient follow-up, the patients were persuaded to comply with the plans. Patients’ compliances were monitored once a week. For those with poor compliance, the frequency of follow-up was increased.	Routine dietary guidance and instruction
Pungchompoo 2019 (Pungchompoo et al., 2020)	SMRP: participants received SMRP in addition to standard care by trained PD nurses which contained structured individualized self-efficacy training program, CBT, self-monitoring skills, self-awareness in goal setting, structured weekly telephone contact, and dialysis-specific education program.	Usual standard care from nursing staff at the renal unit
Wong 2010 (Wong et al., 2010)	Disease Management Program: the participants received both routine care and disease management program. The disease management program was protocol-driven to govern the content and process of the intervention by the team. The team was composed of renal nurses and general nurses. Disease management program included pre-discharge assessment protocol and nurse-initiated telephone call protocols in which nurses would make phone calls to the patient every week for 6 weeks.	Routine care
Xu 2019 (Xu et al., 2020)	I1, Technique inspection: receiving standard initial training and retraining every 2 months in one-on-one sessions by the supervision of nurse. The nurse ensured that each error listed on the NAC form was avoided, and immediately correcting wrong steps I2, Oral education: receiving standard initial training and retraining every 2 months in one-on-one sessions by the supervision of a nurse. The nurse addressed all items on the NAC form one by one to remind the patient of the key points of bag exchange.	Usual care: receiving standard initial training and bag exchange evaluation but did not receive any retraining program.

**Abbreviations**: CBT, cognitive-behavioral therapy; MDT, multidisciplinary team; CLHM, Closed-Loop Health Management; ISPD, international society for peritoneal dialysis; LIP; Liquid Intake Program; NAC, nurse assessment checklist. SMRP, self-management retraining program.

### 3.3 Quality assessment

For the risk of bias assessment of the eleven included studies, nine studies (Chow and Wong, 2010, Hare et al., 2014, Ljungman et al., 2020, Luo et al., 2019, Luo et al., 2020, Wong et al., 2010, Pungchompoo et al., 2020, Li et al., 2021, Chen et al., 2016) had high risk of bias. Two studies had (Chang et al., 2018, Xu et al., 2020) unclear risk of bias. Of the included studies, no studies had overall low risk of bias. A summary of the proportion of included RCTs, which were at low, unclear, and high risk for each risk of bias domain is provided in **Supplementary Material 3, Fig S3.1**. In addition, detailed risk-of-bias assessments for RCTs are provided in **Supplementary Material 3, Figure S3.2.**

### 3.4 Qualitative analysis

#### 3.4.1 Peritonitis

Peritonitis mentioned in two studies could not be included for quantitative analysis (Chang et al., 2018, Li et al., 2021). Chang et al., 2018 reported frequent retraining of PD at home by PD nurses revealed a statistically significant effect on the risk reduction of the first episode of peritonitis. The adjusted HR was 0.01 (95% CI 0.001-0.35, p = 0.01) (Chang et al., 2018). Li et al., 2021 demonstrated a lower incidence of peritonitis in the intervention group compared with the control group (p = 0.008) (Li et al., 2021).

#### 3.4.2 Exit-site infection

One study reported exit-site infection that could not be included in the meta-analysis. Li et al., 2021 reported the lower incidence of exit-site infection in the intervention group in comparison with the control group (p = 0.008.

#### 3.4.3 Quality of life

QoL addressed in the two studies could not be quantitatively analyzed (Hare et al., 2014, Li et al., 2021). Hare et al., 2014. showed no statistically significant change in the adjusted mean of overall QoL using a short-form health survey (SF-36) in the liquid intake program compared with standard care (p = 0.281) (Hare et al., 2014). Li et al., 2021 reported significant improvements in eight domains of SF-36 of the intervention group compared with the control group (Li et al., 2021).

#### 3.4.4 Sleep quality

Of all studies, one study reported on sleep quality that could not be included in the meta-analysis. Chen et al., 2016 demonstrated no statistically significant difference in the median percentages of change in global Pittsburgh Sleep Quality Index scores of the intervention group (cognitive behavioral therapy with sleep hygiene education) compared with the control group (sleep hygiene education alone) (p = 0.3) (Chen et al., 2008).

### 3.5 Quantitative analysis

#### 3.5.1 Peritonitis

**Fig. 2:**
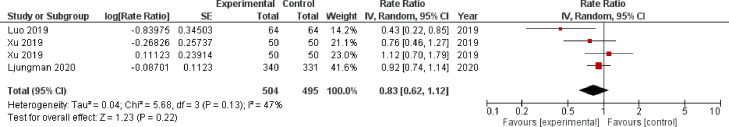
Forest plot of the risk of peritonitis in PD patients.

Three studies reported the risk of peritonitis outcomes (Xu et al., 2020, Ljungman et al., 2020, Luo et al., 2019). One study showed a favorable effect of nurse-based educational intervention (Luo et al., 2019), while other studies reported insufficient evidence on the association between intervention and risk of peritonitis (Xu et al., 2020, Ljungman et al., 2020). Overall, the nurse-based interventions were protective effect against peritonitis, even though not statistically significant (RR 0.83, 95% CI 0.62–1.12) (Fig. 2). Subgroup analysis of peritonitis outcome by intervention type indicated a similar effect with RR 0.83 (95% CI 0.55–1.25) for education-based intervention (Xu et al., 2020, Ljungman et al., 2020, Luo et al., 2019) and RR 0.76 (95% CI 0.46–1.27) for inspection-based intervention **(Supplementary material 4, Figure S4.1)** (Xu et al., 2020).

The figure summarizes the risk of peritonitis of PD patients in three eligible studies. The forest plot represents the pooled estimated rate ratio of peritonitis in PD patients (black diamond). The estimated rate ratio for each study was presented with a red diamond), with 95% confidence intervals (95% CI; horizontal black lines). The overall estimated pooled risk of peritonitis was 0.83 (95% CI 0.62– 1.12). The meta-analysis used a random-effects model with the exact method for confidence interval estimation. *I*^2^, test for heterogeneity. df, degrees of freedom; Z, test of overall treatment effect.

#### 3.5.2 Exit-site infection

Only two studies had exit-site infection outcomes (Chang et al., 2018, Ljungman et al., 2020). Both studies reported no statistically significant association between nurse-based education and exit-site infection. The pooled effect showed no benefit of nurse-based intervention on risk of exit-site infection with RR 0.96 (95% CI 0.76–1.21) as shown in **Supplementary Material 4, Figure S4.2**.

#### 3.5.3 PD-related infection

Only two studies provided PD-related infection outcomes (Chang et al., 2018, Xu et al., 2020). Chang et al., 2018 reported a trend of interventional benefit on PD-related infection (Chang et al., 2018), while Xu et al., 2020 reported no statistically significant benefit of educational intervention on PD-related infection (Xu et al., 2020). Nevertheless, the overall estimate showed a trend of the beneficial effect of nurse educational interventions against PD-related infection with RR 0.72 (95% CI 0.45–1.16) as presented in **Supplementary Material 4, Figure S4.3**.

#### 3.5.4 Hemodialysis transfer

**Fig. 3:**
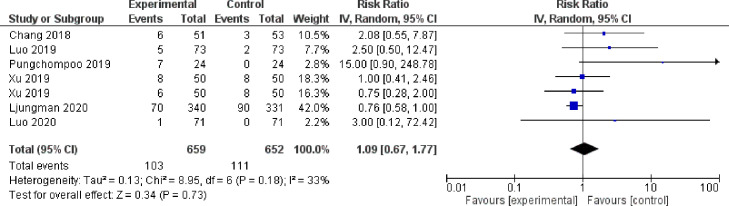
Forest plot of the risk of hemodialysis transfer/technique failure in PD patients.

For HD transfer outcome, six studies were included in the meta-analysis (Chang et al., 2018, Xu et al., 2020, Ljungman et al., 2020, Luo et al., 2019, Luo et al., 2020, Pungchompoo et al., 2020). Ljungman et al., 2020 reported a trend of beneficial effect of the intervention on HD transfer (Ljungman et al., 2020). Meanwhile, two studies demonstrated the opposite trend of negative effect of educational intervention on HD transfer (Luo et al., 2020, Pungchompoo et al., 2020). The pooled estimated effect of nurse-based educational interventions showed no effect on HD transfer (RR 1.09, 95% CI 0.67–1.77) (Fig. 3).

The figure summarizes the risk of HD transfer of PD patients in six eligible studies. The forest plot represents the pooled estimated risk ratio of HD transfer in PD patients (black diamond). The estimated risk ratio for each study was presented with a blue diamond), with 95% confidence intervals (95% CI; horizontal black lines). The overall estimated pooled risk of HD transfer was 1.09 (95% CI 0.67– 1.77). The meta-analysis used a random-effects model with the exact method for confidence interval estimation. *I*^2^, test for heterogeneity. df, degrees of freedom; Z, test of overall treatment effect.

#### 3.5.5 Mortality

There were six studies (Chang et al., 2018, Xu et al., 2020, Chow and Wong, 2010, Ljungman et al., 2020, Luo et al., 2020, Wong et al., 2010) included for meta-analysis of mortality outcomes (Chang et al., 2018, Xu et al., 2020, Chow and Wong, 2010, Ljungman et al., 2020, Luo et al., 2020, Wong et al., 2010). All six studies showed insufficient evidence of the association between nurse-based interventions and mortality. An overall risk ratio for mortality was 1.06 (95% CI 0.76–1.47), which indicated no beneficial effect of the educational interventions on mortality **SupplementaryMaterial 4, Figure S4.4**.

#### 3.5.6 Overall quality of life

**Fig. 4:**
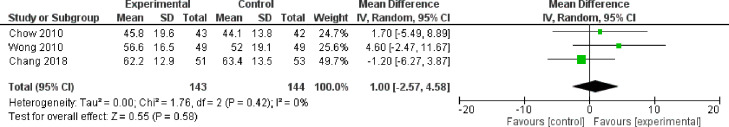
Forest plot of the overall QoL in PD patients.

Three studies provided information on the overall QoL assessed by Kidney Disease Quality of Life (KDQoL) (Chang et al., 2018, Chow and Wong, 2010, Wong et al., 2010). None of the three studies provided evidence of the association between nurse-based educational interventions and overall QoL in PD patients. The pooled estimated mean difference was 1.00 (95% CI -2.75–4.58), which showed no statistically significant difference between the intervention and control groups (Fig. 4).

The figure summarizes the overall QoL of PD patients in three eligible studies. The forest plot represents the pooled estimated mean difference of overall QoL in PD patients (black diamond). The estimated mean difference of treatment group compared to control group for each study was presented with a green diamond), with 95% confidence intervals (95% CI; horizontal black lines). The overall estimated pooled QoL was 1.00 (95% CI -2.57– 4.58). The meta-analysis used a random-effects model with the exact method for confidence interval estimation. *I*^2^, test for heterogeneity. df, degrees of freedom; Z, test of overall treatment effect.

#### 3.5.7 Sleep quality

**Fig. 5:**
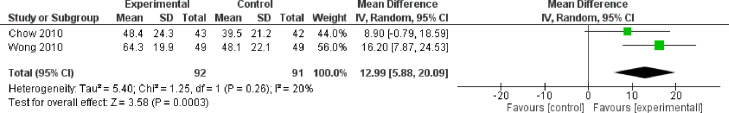
Forest plot of the sleep quality in PD patients.

Only two studies had information on sleep quality assessed by KDQoL (Chow and Wong, 2010, Wong et al., 2010). Wong et al., 2010 showed a statistically significant benefit of nurse-based educational intervention on sleep quality (Wong et al., 2010), while Chow et al., 2010 presented a similar beneficial trend, although non-significant (Chow and Wong, 2010). Overall, there was a statistically significant effect of educational interventions on sleep quality, which pooled mean difference was 12.76 (95% CI 5.26–20.27) (Fig. 5).

The figure summarizes the sleep quality of PD patients in two eligible studies. The forest plot represents the pooled estimated mean difference of the sleep QoL in PD patients (black diamond). The estimated mean difference of treatment group compared to control group for each study was presented with a green diamond), with 95% confidence intervals (95% CI; horizontal black lines). The overall estimated pooled sleep quality was 12.99 (95% CI 5.88– 20.09). The meta-analysis used a random-effects model with the exact method for confidence interval estimation. *I*^2^, test for heterogeneity. df, degrees of freedom; Z, test of overall treatment effect.

### 3.6 Sensitivity analysis

For quantitative analyses of more than two included studies, we performed the sensitivity analysis by excluding one study that had a different methodology at a time. The results were similar to the primary analyses on the peritonitis rates, HD transfer event rates, mortality rates, and overall QoL.

### 3.7 Funnel plot

The funnel plot was not conducted due to fewer than ten included studies included in the meta-analysis of the primary outcomes. As a result, possibilities of potential publication bias could not be ruled out.

## 4 Discussion

To our knowledge, this study was the first systematic review and meta-analysis of RCTs focusing on nurse-based educational interventions in ESKD patients receiving PD. The pooled data of the study found the only advantage on sleep quality from nurse-based educational interventions but no statistical significance in the other outcomes. Nevertheless, there was a trend that nurse-based educational interventions could reduce the peritonitis risk (RR 0.83, 95% CI 0.62–1.12). In addition, from the qualitative analysis, Chang et al., 2016 revealed that frequent retraining of PD at home by PD nurses statistically significantly reduced adjusted hazard ratio of the first episode of peritonitis (HR = 0.01, 95 percent CI 0.001-0.35) (Chang et al., 2018). The exit-site and PD-related infections were also not statistically significant in our studies. However, the results of exit-site infections and PD-related infections were supported by two studies (Chang et al., 2018, Ljungman et al., 2020) and two studies (Chang et al., 2018, Xu et al., 2020), respectively.

From the included trials (Chang et al., 2018, Xu et al., 2020, Ljungman et al., 2020, Luo et al., 2019), we possibly imply that more efficacious education and contact time of nurses given to PD patients could lead to more protection against peritonitis, exit-site infections, and PD-related infections. Concerning sleep quality, statistical significance was revealed. As sleep disturbance often occurred in ESKD patients (Perl et al., 2006, Tang and Lai, 2009, Hui et al., 2000), sleep quality in PD patients was known to decline by the time (Masoumi et al., 2013, Unruh et al., 2006, Unruh et al., 2003). The beneficial effect of the nurse-based intervention on improving sleep quality in PD patients is consistent with the finding in patients with chronic kidney disease (CKD) (Chen et al., 2016) and is possibly related to multiple mechanisms, including improvement of malnutrition-inflammation status (Lai et al., 2015, Azarnoush et al., 2021), timely detection of complications (Scherpbier-de Haan et al., 2013), and relieving in anxiety or depression (Lui et al., 2002, Steele et al., 1996). An inflammatory state has been associated to decreased sleep quality in dialysis (PD) patients (Unruh et al., 2006, Chiu et al., 2009). Malnutrition has been also linked with lower sleep quality in PD patients (Li et al., 2012). The proposed mechanism was malnutrition changes hormonal regulation and raises serotonin levels in the brain, which increases slow-wave sleep and contributes to poor sleep quality (Mokler et al., 1999).

Several meta-analyses studies on interventions purposed to reduce the risks of peritonitis and exit-site infections in PD patients have been published (Htay et al., 2019, Strippoli et al., 2004, Campbell et al., 2017). In the Cochrane reviews, the catheter-related interventions, such as the use of different catheter types and different insertion techniques, demonstrated no benefit in reducing the risk of peritonitis and exit-site infections (Htay et al., 2019, Strippoli et al., 2004). The use of nasal, oral, or topical antibiotics also had uncertain effects on the risk of peritonitis and exit-site/tunnel infections compared with placebo or no treatment (Campbell et al., 2017). Similar to our study on nurse-based educational interventions, the numbers and sizes of recruited studies were small. Moreover, the methodological quality of the studies was suboptimal. Therefore, the plausibility that nurse-based interventions could have a beneficial effect on reducing peritonitis or exit-site infections cannot be completely ruled out with confidence. Further RCTs are needed to verify the effectiveness of nurse-based educational interventions in reducing the PD-related peritonitis rates.

Nurse-led interventions and models seemed to have potential in various situations (Deschodt et al., 2020, Li et al., 2020, Chavez et al., 2018, Randall et al., 2017). One study on a nurse-coordinated model of care reduced the risks of composite death, ESKD, and doubling of serum creatinine when compared with the usual care group in CKD patients (Xu et al., 2017). In congestive heart failure (CHF), nurse-led heart failure self-care education significantly reduces the risk of all-cause readmission and CHF-specific readmission (Son et al., 2020). Nurse-led interventions also demonstrated improvement on blood pressure and glycemic control in people with hypertension and diabetes (Clark et al., 2010, Tshiananga et al., 2011) .

Moreover, nurse-led interventions were reported to enhance adherence to chronic medications (Van Camp et al., 2013). As poor self-care and behaviors could increase the risk of adverse outcomes and complications in chronic diseases, nurse-based interventions showed more solid evidence on the improvement of objective outcomes and the prevention of adverse events. These findings perhaps implicate that nurse-based interventions in PD patients could participate in diet restriction, improvement of self- hygiene, and enhancement of dialysis adherence. However, there are still gaps of knowledge needed to be filled by studies on the efficacy of nurse-based educational interventions on PD patients in a variety of nursing settings, such as nurse-coordinated education, virtual educational training, and comprehensive telemedicine.

The major limitation of our study was the number and size of included studies. Although certain non-English articles might be relevant, we were unable to identify these articles. Of eleven studies, only three studies were included in the meta-analysis of the peritonitis rate. Moreover, the risk of bias in this systematic review was high in the eight studies (Chow and Wong, 2010, Hare et al., 2014, Li et al., 2021,Ljungman et al., 2020, Luo et al., 2019, Luo et al., 2020, Wong et al., 2010, Pungchompoo et al., 2020) from the eleven studies (Chang et al., 2018, Xu et al., 2020, Chow and Wong, 2010, Hare et al., 2014, Ljungman et al., 2020, Luo et al., 2019, Li et al., 2021,Luo et al., 2020, Wong et al., 2010, Pungchompoo et al., 2020, Chen et al., 2016). This is probably due to the nature of the interventions, which were problematic to blind participants, intervention delivers, and outcome investigators. Thus, all of the eleven studies (Chang et al., 2018, Xu et al., 2020, Chow and Wong, 2010, Hare et al., 2014, Ljungman et al., 2020, Luo et al., 2019, Li et al., 2021,Luo et al., 2020, Wong et al., 2010, Pungchompoo et al., 2020, Chen et al., 2016) were open-label trials. Recently, because there had been general improvements in peritonitis rates globally (Li et al., 2017, Mehrotra et al., 2016), the improvement of peritonitis rates from interventions compared to standard care might be potentially difficult to achieve statistical significance. The long-term follow-up period possibly helps demonstrate more differences in peritonitis rates, PD-related infections, HD transfer, and mortality between intervention and control groups (De Sousa-Amorim et al., 2013, Tekkarişmaz and Torun, 2020, Vikrant, 2014, Han et al., 2008). For example, 367 people out of 541 in one research experienced their first peritonitis episode within 12 months after taking PD, indicating that patients should be followed for at least 12 months (van Diepen et al., 2015). Moreover, publication bias could not be evaluated due to less than ten studies included in the meta-analysis. Hence, more evidence seems to be needed to validate the study results.

## 5 Conclusions

Nurse-based educational interventions potentially help reduce certain PD complications in PD patients, of which only the sleep quality showed statistically significant improvement in the study. However, evidence on the nurse-based educational interventions in patients receiving PD was limited. Additional high-quality RCTs of nurse-based intervention in PD patients with adequate follow-up time are still needed to provide more robust effectiveness in peritonitis, PD-related infections, technique failure, QoL, and mortality.

## Funding sources

This study was partly supported by the Thailand Science Research and Innovation Fund Chulalongkorn University (CU_FRB65_hea (19)_026_30_07), Chulalongkorn University, 10.13039/501100004704National Research Council of Thailand (6/2562).

## CRediT authorship contribution statement

**Tanawin Nopsopon:** Data curation, Formal analysis, Investigation, Methodology, Visualization, Writing – original draft, Writing – review & editing. **Piyawat Kantagowit:** Data curation, Formal analysis, Investigation, Visualization, Writing – review & editing. **Chitsanucha Chumsri:** Data curation, Investigation, Writing – review & editing. **Piyaporn Towannang:** Data curation, Investigation. **Apinya Wechpradit:** Data curation, Investigation. **Nipa Aiyasanon:** Data curation, Investigation. **Ruchdaporn Phaichan:** Data curation, Investigation. **Talerngsak Kanjanabuch:** Conceptualization, Project administration, Resources, Supervision, Validation, Writing – review & editing. **Krit Pongpirul:** Conceptualization, Funding acquisition, Methodology, Project administration, Resources, Supervision, Validation, Writing – original draft, Writing – review & editing.

## Declaration of Competing Interest

T.K. has received consultancy fees from VISTERRA as a country investigator and current recipient of the National Research Council of Thailand and the Thailand Science Research and Innovation Fund Chulalongkorn University, Thailand and received speaking honoraria from Astra Zeneca and Baxter Healthcare.

## Appendix Supplementary materials

Image, application 1

Image, application 2

Image, application 3

## References

[bib0001] Azarnoush H, Mortazavi M, Rouhani MH (2021). Dietary nutrients' intake and sleep quality in peritoneal dialysis patients. Sleep Science (Sao Paulo, Brazil).

[bib0002] Campbell D, Mudge DW, Craig JC, Johnson DW, Tong A, Strippoli GF. (2017). Antimicrobial agents for preventing peritonitis in peritoneal dialysis patients. Cochrane Database Syst. Rev..

[bib0003] Chang JH, Oh J, Park SK (2018). Frequent patient retraining at home reduces the risks of peritoneal dialysis-related infections: a randomised study. Sci. Rep..

[bib0004] Chang YT, Hwang JS, Hung SY (2016). Cost-effectiveness of hemodialysis and peritoneal dialysis: a national cohort study with 14 years follow-up and matched for comorbidities and propensity score. Sci. Rep..

[bib0005] Chavez KS, Dwyer AA, Ramelet AS. (2018). International practice settings, interventions and outcomes of nurse practitioners in geriatric care: a scoping review. Int. J. Nurs. Stud..

[bib0006] Chen CC, Chen Y, Liu X (2016). The efficacy of a nurse-led disease management program in improving the quality of life for patients with chronic kidney disease: a meta-analysis. PLoS One.

[bib0007] Chen HY, Chiang CK, Wang HH (2008). Cognitive-behavioral therapy for sleep disturbance in patients undergoing peritoneal dialysis: a pilot randomized controlled trial. Am. J. Kidney Dis..

[bib0008] Chiu YL, Chuang YF, Fang KC (2009). Higher systemic inflammation is associated with poorer sleep quality in stable haemodialysis patients. Nephrol. Dial. Transplant..

[bib0009] Chow SK, Wong FK. (2010). Health-related quality of life in patients undergoing peritoneal dialysis: effects of a nurse-led case management programme. J. Adv. Nurs..

[bib0010] Clark CE, Smith LF, Taylor RS, Campbell JL. (2010). Nurse led interventions to improve control of blood pressure in people with hypertension: systematic review and meta-analysis. BMJ.

[bib0011] Deschodt M, Laurent G, Cornelissen L (2020). Core components and impact of nurse-led integrated care models for home-dwelling older people: a systematic review and meta-analysis. Int. J. Nurs. Stud..

[bib0012] De Sousa-Amorim E, Bajo-Rubio MA, del Peso-Gilsanz G, Castro MJ, Celadilla O, Selgas-Gutiérrez R. (2013). Thirty years in a peritoneal dialysis unit: long-term survival. Nefrologia.

[bib0013] Dong J, Chen Y. (2010). Impact of the bag exchange procedure on risk of peritonitis. Perit. Dial. Int..

[bib0014] Ferguson TW, Whitlock RH, Bamforth RJ (2021). Cost-utility of dialysis in Canada: hemodialysis, peritoneal dialysis, and nondialysis treatment of kidney failure. Kidney Med..

[bib0015] Han SH, Lee JE, Kim DK (2008). Long-term clinical outcomes of peritoneal dialysis patients: single center experience from Korea. Perit. Dial. Int..

[bib0016] Hare J, Clark-Carter D, Forshaw M. (2014). A randomized controlled trial to evaluate the effectiveness of a cognitive behavioural group approach to improve patient adherence to peritoneal dialysis fluid restrictions: a pilot study. Nephrol. Dial. Transplant..

[bib0017] Higgins J, Thomas J, Chandler J (2019).

[bib0018] Htay H, Johnson DW, Craig JC (2019). Catheter type, placement and insertion techniques for preventing catheter-related infections in chronic peritoneal dialysis patients. Cochrane Database Syst. Rev..

[bib0019] Hui DS, Wong TY, Ko FW (2000). Prevalence of sleep disturbances in chinese patients with end-stage renal failure on continuous ambulatory peritoneal dialysis. Am. J. Kidney Dis..

[bib0020] Karopadi AN, Mason G, Rettore E, Ronco C. (2013). Cost of peritoneal dialysis and haemodialysis across the world. Nephrol. Dial. Transplant..

[bib0021] Lai X, Chen W, Bian X (2015). Predictors of poor sleep quality and excessive daytime sleepiness in peritoneal dialysis patients. Renal Failure.

[bib0022] Li C, Liu Y, Xue D, Chan CWH. (2020). Effects of nurse-led interventions on early detection of cancer: a systematic review and meta-analysis. Int. J. Nurs. Stud..

[bib0023] Li F, Wang Y, Shi S. (2021). Observation of the effect of closed-loop health management based on an internet platform in patients with peritoneal dialysis: a randomized trial. Ann. Palliat. Med..

[bib0024] Li J, Guo Q, Ye X (2012). Prevalence and risk factors of sleep disturbance in continuous ambulatory peritoneal dialysis patients in Guangzhou, southern China. Int. Urol. Nephrol..

[bib0025] Li PK, Chow KM, Van de Luijtgaarden MW (2017). Changes in the worldwide epidemiology of peritoneal dialysis. Nat. Rev. Nephrol..

[bib0026] Li PK, Chow KM. (2013). Peritoneal dialysis-first policy made successful: perspectives and actions. Am. J. Kidney Dis..

[bib0027] Ljungman S, Jensen JE, Paulsen D (2020). Retraining for prevention of peritonitis in peritoneal dialysis patients: a randomized controlled trial. Perit. Dial. Int..

[bib0028] Lui SL, Ng F, Lo WK. (2002). Factors associated with sleep disorders in Chinese patients on continuous ambulatory peritoneal dialysis. Perit. Dial. Int..

[bib0029] Luo Y, Huang Y, Chen X, Meng G, Zhang Y. (2019). Effects of multidisciplinary team care based on 5E's renal rehabilitation for peritoneal dialysis patients in Guangxi Zhuang autonomous region of China: a randomized controlled trial. Blood. Purif..

[bib0030] Luo Y, Huang Y, Zhang Y, Xiang J, Wu Q. (2020). Effect of nurse-led food exchange intervention for patients undergoing peritoneal dialysis. Clin. Nephrol..

[bib0031] Manera KE, Johnson DW, Craig JC (2020). Establishing a core outcome set for peritoneal dialysis: report of the SONG-PD (Standardized Outcomes in Nephrology-Peritoneal Dialysis) consensus workshop. Am. J. Kidney Dis..

[bib0032] Masoumi M, Naini AE, Aghaghazvini R, Amra B, Gholamrezaei A. (2013). Sleep quality in patients on maintenance hemodialysis and peritoneal dialysis. Int. J. Prev. Med..

[bib0033] Mathew AT, Fishbane S, Obi Y, Kalantar-Zadeh K. (2016). Preservation of residual kidney function in hemodialysis patients: reviving an old concept. Kidney Int..

[bib0034] Mawar S, Gupta S, Mahajan S. (2012). Non-compliance to the continuous ambulatory peritoneal dialysis procedure increases the risk of peritonitis. Int. Urol. Nephrol..

[bib0035] Mehrotra R, Devuyst O, Davies SJ, Johnson DW. (2016). The current state of peritoneal dialysis. J. Am. Soc. Nephrol..

[bib0036] Misra M, Vonesh E, Van Stone JC, Moore HL, Prowant B, Nolph KD. (2001). Effect of cause and time of dropout on the residual GFR: a comparative analysis of the decline of GFR on dialysis. Kidney Int..

[bib0037] Moist LM, Port FK, Orzol SM (2000). Predictors of loss of residual renal function among new dialysis patients. J. Am. Soc. Nephrol..

[bib0038] Mokler DJ, Bronzino JD, Galler JR, Morgane PJ. (1999). The effects of median raphé electrical stimulation on serotonin release in the dorsal hippocampal formation of prenatally protein malnourished rats. Brain Res..

[bib0039] Perl J, Unruh ML, Chan CT. (2006). Sleep disorders in end-stage renal disease: 'Markers of inadequate dialysis'?. Kidney Int..

[bib0040] Pungchompoo W, Parinyajittha S, Pungchompoo S, Kumtan P. (2020). Effectiveness of a self-management retraining program improving the quality of life of people receiving continuous ambulatory peritoneal dialysis. Nurs. Health Sci..

[bib0041] Randall S, Crawford T, Currie J, River J, Betihavas V. (2017). Impact of community based nurse-led clinics on patient outcomes, patient satisfaction, patient access and cost effectiveness: a systematic review. Int. J. Nurs. Stud..

[bib0042] Review Manager (2014). Copenhagen: Nordic Cochrane Centre.

[bib0043] Rubin HR, Fink NE, Plantinga LC, Sadler JH, Kliger AS, Powe NR. (2004). Patient ratings of dialysis care with peritoneal dialysis vs hemodialysis. JAMA.

[bib0044] Scherpbier-de Haan ND, Vervoort GMM, van Weel C (2013). Effect of shared care on blood pressure in patients with chronic kidney disease: a cluster randomised controlled trial. Br. J. Gen. Pract. J. Royal Coll. Gen. Pract..

[bib0045] Shamseer L, Moher D, Clarke M (2015). Preferred reporting items for systematic review and meta-analysis protocols (PRISMA-P) 2015: elaboration and explanation. BMJ.

[bib0046] Son YJ, Choi J, Lee HJ. (2020). Effectiveness of nurse-led heart failure self-care education on health outcomes of heart failure patients: a systematic review and meta-analysis. Int. J. Environ. Res. Public Health.

[bib0047] Steele TE, Baltimore D, Finkelstein SH, Juergensen P, Kliger AS, Finkelstein FO. (1996). Quality of life in peritoneal dialysis patients. J. Nerv. Ment. Dis..

[bib0048] Sterne J, Savović J, Page M (2019). RoB 2: a revised tool for assessing risk of bias in randomised trials. BMJ.

[bib0049] Sterne JAC, Sutton AJ, Ioannidis JPA (2011). Recommendations for examining and interpreting funnel plot asymmetry in meta-analyses of randomised controlled trials. BMJ.

[bib0050] Strippoli GF, Tong A, Johnson D, Schena FP, Craig JC. (2004). Catheter type, placement and insertion techniques for preventing peritonitis in peritoneal dialysis patients. Cochrane Database Syst. Rev..

[bib0051] Tang SC, Lai KN. (2009). Sleep disturbances and sleep apnea in patients on chronic peritoneal dialysis. J. Nephrol..

[bib0052] Tekkarişmaz N, Torun D. (2020). Long-term clinical outcomes of peritoneal dialysis patients: 9-year experience of a single center in Turkey. Turkish J. Med. Sci..

[bib0053] Tshiananga JKT, Kocher S, Weber C, Erny-Albrecht K, Berndt K, Neeser K (2011). The effect of nurse-led diabetes self-management education on glycosylated hemoglobin and cardiovascular risk factors: a meta-analysis. The Diabetes Educator.

[bib0054] Unruh ML, Buysse DJ, Dew MA (2006). Sleep quality and its correlates in the first year of dialysis. Clin. J. Am. Soc. Nephrol..

[bib0055] Unruh ML, Hartunian MG, Chapman MM, Jaber BL. (2003). Sleep quality and clinical correlates in patients on maintenance dialysis. Clin. Nephrol..

[bib0056] Van Camp YP, Van Rompaey B, Elseviers MM. (2013). Nurse-led interventions to enhance adherence to chronic medication: systematic review and meta-analysis of randomised controlled trials. Eur. J. Clin. Pharmacol..

[bib0057] van Diepen AT, van Esch S, Struijk DG, Krediet RT. (2015). The first peritonitis episode alters the natural course of peritoneal membrane characteristics in peritoneal dialysis patients. Perit. Dial. Int..

[bib0058] Vikrant S. (2014). Long-term clinical outcomes of peritoneal dialysis patients: 9-year experience of a single center from north India. Perit. Dial. Int..

[bib0059] Wong B, Ravani P, Oliver MJ (2018). Comparison of patient survival between hemodialysis and peritoneal dialysis among patients eligible for both modalities. Am. J. Kidney Dis..

[bib0060] Wong FK, Chow SK, Chan TM. (2010). Evaluation of a nurse-led disease management programme for chronic kidney disease: a randomized controlled trial. Int. J. Nurs. Stud..

[bib0061] Xu H, Mou L, Cai Z. (2017). A nurse-coordinated model of care versus usual care for chronic kidney disease: meta-analysis. J. Clin. Nurs..

[bib0062] Xu Y, Zhang Y, Yang B (2020). Prevention of peritoneal dialysis-related peritonitis by regular patient retraining via technique inspection or oral education: a randomized controlled trial. Nephrol. Dial. Transplant..

