# Supplementary material for: Nurse-based educational interventions in patients with peritoneal dialysis: A systematic review and meta-analysis
Source: Int J Nurs Stud Adv. 2022 Sep 24;4:100102. doi: 10.1016/j.ijnsa.2022.100102 (PMC11080474; doi:10.1016/j.ijnsa.2022.100102)
Supplement: Supplementary file 1 [file mmc1.docx]

**Supplementary material 2**

“Search strategy for Nurse-based Patient Education for Peritoneal Dialysis: A systematic review and meta-analysis”

Concept 1. Peritoneal Dialysis

1.1 Not Parkinson’s Disease

2. Patient Education

3. RCT

***PubMed***

| Set # | PubMed till Feb 20, 2020 | Results |
| --- | --- | --- |
| 1  Peritoneal Dialysis | "peritoneal dialysis"[MeSH Terms] OR (peritoneal[tiab] AND (dialysis[tiab] OR dialyses[tiab])) OR "peritoneal dialysis"[tiab] OR "peritoneal dialyses"[tiab] OR pd[tiab] OR capd[tiab] OR ccpd[tiab] OR apd[tiab] | **156935** |
| 2  Parkinson Disease | "parkinson disease"[MeSH Terms] OR parkinson*[tiab] | **121641** |
| 3 | #1 NOT #2 | **114459** |
| 4  Patient Education | "patient education as topic"[MeSH Terms] OR "health education"[MeSH Terms] OR ((patient*[tiab] OR health*[tiab]) AND educat*[tiab]) OR "patient education"[tiab] OR "health education"[tiab] OR (patient*[tiab] AND (communicat*[tiab] OR interacti*[tiab] OR inform*[tiab] OR instruct*[tiab] OR advice*[tiab] OR advise*[tiab] OR counsel*[tiab] OR consel*[tiab] OR teach*[tiab] OR train*[tiab] OR empower*[tiab])) OR "patient communication"[tiab] OR "patient counseling"[tiab] OR (educat*[tiab] AND (intervention*[tiab] OR rehabilitation*[tiab] OR program*[tiab] OR service*[tiab] OR group*[tiab] OR session*[tiab])) OR leaflet*[tiab] OR booklet*[tiab] OR pamphlet*[tiab] OR poster*[tiab] OR video*[tiab] OR ((written[tiab] OR printed[tiab] OR oral[tiab]) AND information*[tiab]) OR "academic detailing"[tiab] OR (training[tiab] AND program*[tiab]) OR algorithm*[tiab] OR (decision[tiab] AND tree*[tiab]) OR (self[tiab] AND care*[tiab]) OR selfcare[tiab] OR "self-care"[tiab] | **2120643** |
| 5  RCT | "randomized controlled trial"[pt] OR trial[tiab] OR randomized[tiab] OR randomised[tiab] OR randomly[tiab] OR placebo[tiab] OR groups[tiab] OR "drug therapy"[sh] | **4676696** |
| 6 | #3 AND #4 AND #5 | **2633** |
| 7 | animals[MeSH Terms] NOT humans[MeSH Terms] | **4669162** |
| 8 | #6 NOT #7 | **2570** |
| 9 | English[lang] | **25949157** |
| 10 | #8 AND #9 | **2435** |

| Set # | PubMed from Feb 21, 2020, to Dec 31, 2021 | Results |
| --- | --- | --- |
| 1  Peritoneal Dialysis | "peritoneal dialysis"[MeSH Terms] OR (peritoneal[tiab] AND (dialysis[tiab] OR dialyses[tiab])) OR "peritoneal dialysis"[tiab] OR "peritoneal dialyses"[tiab] OR pd[tiab] OR capd[tiab] OR ccpd[tiab] OR apd[tiab] | **200353** |
| 2  Parkinson Disease | "parkinson disease"[MeSH Terms] OR parkinson*[tiab] | **143363** |
| 3 | #1 NOT #2 | **146660** |
| 4  Patient Education | "patient education as topic"[MeSH Terms] OR "health education"[MeSH Terms] OR ((patient*[tiab] OR health*[tiab]) AND educat*[tiab]) OR "patient education"[tiab] OR "health education"[tiab] OR (patient*[tiab] AND (communicat*[tiab] OR interacti*[tiab] OR inform*[tiab] OR instruct*[tiab] OR advice*[tiab] OR advise*[tiab] OR counsel*[tiab] OR consel*[tiab] OR teach*[tiab] OR train*[tiab] OR empower*[tiab])) OR "patient communication"[tiab] OR "patient counseling"[tiab] OR (educat*[tiab] AND (intervention*[tiab] OR rehabilitation*[tiab] OR program*[tiab] OR service*[tiab] OR group*[tiab] OR session*[tiab])) OR leaflet*[tiab] OR booklet*[tiab] OR pamphlet*[tiab] OR poster*[tiab] OR video*[tiab] OR ((written[tiab] OR printed[tiab] OR oral[tiab]) AND information*[tiab]) OR "academic detailing"[tiab] OR (training[tiab] AND program*[tiab]) OR algorithm*[tiab] OR (decision[tiab] AND tree*[tiab]) OR (self[tiab] AND care*[tiab]) OR selfcare[tiab] OR "self-care"[tiab] | **2602224** |
| 5  RCT | "randomized controlled trial"[pt] OR trial[tiab] OR randomized[tiab] OR randomised[tiab] OR randomly[tiab] OR placebo[tiab] OR groups[tiab] OR "drug therapy"[sh] | **5435377** |
| 6 | #3 AND #4 AND #5 | **4097** |
| 7 | animals[MeSH Terms] NOT humans[MeSH Terms] | **5014209** |
| 8 | #6 NOT #7 | **4007** |
| 9 | English[lang] | **29410527** |
| 10 | #8 AND #9 | **3848** |
| 11 | 2020/02/21:2021/12/31[dp] | **2948605** |
| 12 | #10 AND #11 | **1374** |

***Embase***

| Set # | Embase till Feb 20, 2020 | Results |
| --- | --- | --- |
| 1  Peritoneal Dialysis | 'peritoneal dialysis'/exp OR ('peritoneal':ti,ab AND ('dialysis':ti,ab OR 'dialyses':ti,ab)) OR 'peritoneal dialysis':ti,ab OR 'peritoneal dialyses':ti,ab OR 'pd':ti,ab OR 'capd':ti,ab OR 'ccpd':ti,ab OR 'apd':ti,ab | **251753** |
| 2  Parkinson  Disease | 'parkinson disease'/exp OR 'parkinson*':ti,ab | **194285** |
| 3 | #1 NOT #2 | **182195** |
| 4  Patient  Education | 'patient education'/exp OR 'health education'/exp OR (('patient*' OR 'health*') NEAR/6 'educat*'):ti,ab OR 'patient education':ti,ab OR 'health education':ti,ab OR ('patient*' NEAR/6 ('communicat*' OR 'interacti*' OR 'inform*' OR 'instruct*' OR 'advice*' OR 'advise*' OR 'counsel*' OR 'consel*' OR 'teach*' OR 'train*' OR 'empower*')):ti,ab OR 'patient communication':ti,ab OR 'patient counseling':ti,ab OR ('educat*' NEAR/6 ('intervention*' OR 'rehabilitation*' OR 'program*' OR 'service*' OR 'group*' OR 'session*')):ti,ab OR 'leaflet*':ti,ab OR 'booklet*':ti,ab OR 'pamphlet*':ti,ab OR 'poster*':ti,ab OR 'video*':ti,ab OR (('written' OR 'printed' OR 'oral') NEAR/3 'information*'):ti,ab OR 'academic detailing':ti,ab OR ('training' NEXT 'program*'):ti,ab OR 'algorithm*':ti,ab OR ('decision' NEXT 'tree*'):ti,ab OR ('self' NEXT 'care*'):ti,ab OR 'selfcare':ti,ab OR 'self-care':ti,ab | **1743463** |
| 5  RCT | 'randomized controlled trial'/exp OR 'randomization'/exp OR 'double blind procedure'/exp OR 'single blind procedure'/exp OR 'random*':ti,ab OR 'clinical trial'/exp OR ('clin*' NEAR/3 'trial*'):ti,ab OR (('singl*' OR 'doubl*' OR 'trebl*' OR 'tripl*') NEAR/3 ('blind*' OR 'mask*')):ti,ab OR 'placebo'/exp OR 'placebo*':ti,ab OR 'experimental design'/exp OR 'crossover procedure'/exp OR 'control group'/exp OR 'latin square design'/exp OR 'comparative study'/exp OR 'evaluation'/exp OR 'prospective study'/exp OR ('control*' OR 'prospectiv*' OR 'volunteer*'):ti,ab | **8603687** |
| 6 | #3 AND #4 AND #5 | **3521** |
| 7 | [animals]/lim NOT [humans]/lim | **5749265** |
| 8 | #6 NOT #7 | **3354** |
| 9 | english:la | **30835401** |
| 10 | #8 AND #9 | **3246** |

| Set # | Embase from Feb 21, 2020, to Dec 31, 2021 | Results |
| --- | --- | --- |
| 1  Peritoneal Dialysis | 'peritoneal dialysis'/exp OR ('peritoneal':ti,ab AND ('dialysis':ti,ab OR 'dialyses':ti,ab)) OR 'peritoneal dialysis':ti,ab OR 'peritoneal dialyses':ti,ab OR 'pd':ti,ab OR 'capd':ti,ab OR 'ccpd':ti,ab OR 'apd':ti,ab | **322476** |
| 2  Parkinson  Disease | 'parkinson disease'/exp OR 'parkinson*':ti,ab | **231151** |
| 3 | #1 NOT #2 | **235806** |
| 4  Patient  Education | 'patient education'/exp OR 'health education'/exp OR (('patient*' OR 'health*') NEAR/6 'educat*'):ti,ab OR 'patient education':ti,ab OR 'health education':ti,ab OR ('patient*' NEAR/6 ('communicat*' OR 'interacti*' OR 'inform*' OR 'instruct*' OR 'advice*' OR 'advise*' OR 'counsel*' OR 'consel*' OR 'teach*' OR 'train*' OR 'empower*')):ti,ab OR 'patient communication':ti,ab OR 'patient counseling':ti,ab OR ('educat*' NEAR/6 ('intervention*' OR 'rehabilitation*' OR 'program*' OR 'service*' OR 'group*' OR 'session*')):ti,ab OR 'leaflet*':ti,ab OR 'booklet*':ti,ab OR 'pamphlet*':ti,ab OR 'poster*':ti,ab OR 'video*':ti,ab OR (('written' OR 'printed' OR 'oral') NEAR/3 'information*'):ti,ab OR 'academic detailing':ti,ab OR ('training' NEXT 'program*'):ti,ab OR 'algorithm*':ti,ab OR ('decision' NEXT 'tree*'):ti,ab OR ('self' NEXT 'care*'):ti,ab OR 'selfcare':ti,ab OR 'self-care':ti,ab | **2134173** |
| 5  RCT | 'randomized controlled trial'/exp OR 'randomization'/exp OR 'double blind procedure'/exp OR 'single blind procedure'/exp OR 'random*':ti,ab OR 'clinical trial'/exp OR ('clin*' NEAR/3 'trial*'):ti,ab OR (('singl*' OR 'doubl*' OR 'trebl*' OR 'tripl*') NEAR/3 ('blind*' OR 'mask*')):ti,ab OR 'placebo'/exp OR 'placebo*':ti,ab OR 'experimental design'/exp OR 'crossover procedure'/exp OR 'control group'/exp OR 'latin square design'/exp OR 'comparative study'/exp OR 'evaluation'/exp OR 'prospective study'/exp OR ('control*' OR 'prospectiv*' OR 'volunteer*'):ti,ab | **10095019** |
| 6 | #3 AND #4 AND #5 | **4857** |
| 7 | [animals]/lim NOT [humans]/lim | **6407416** |
| 8 | #6 NOT #7 | **4664** |
| 9 | english:la | **35628055** |
| 10 | #8 AND #9 | **4534** |
| 11 | [21-02-2020]/sd | **4973893** |
| 12 | [01-01-2022]/sd | **1274889** |
| 13 | #11 NOT #12 | **3699004** |
| 14 | #10 AND #13 | **992** |

***Cochrane Central Register of Controlled Trials (CENTRAL)***

| Set # | CENTRAL till Feb 20, 2020 | Results |
| --- | --- | --- |
| 1  Peritoneal Dialysis | [mh "peritoneal dialysis"] OR (peritoneal:ti,ab,kw AND (dialysis:ti,ab,kw OR dialyses:ti,ab,kw)) OR "peritoneal dialysis":ti,ab,kw OR "peritoneal dialyses":ti,ab,kw OR pd:ti,ab,kw OR capd:ti,ab,kw OR ccpd:ti,ab,kw OR apd:ti,ab,kw | **36699** |
| 2  Parkinson  Disease | [mh "parkinson disease"] OR parkinson*:ti,ab,kw | **10322** |
| 3 | #1 NOT #2 | **31821** |
| 4  Patient Education | [mh "patient education as topic"] OR [mh "health education"] OR ((patient* OR health*) near/6 educat*):ti,ab,kw OR "patient education":ti,ab,kw OR "health education":ti,ab,kw OR (patient* near/6 (communicat* OR interacti* OR inform* OR instruct* OR advice* OR advise* OR counsel* OR consel* OR teach* OR train* OR empower*)):ti,ab,kw OR "patient communication":ti,ab,kw OR "patient counseling":ti,ab,kw OR (educat* near/6 (intervention* OR rehabilitation* OR program* OR service* OR group* OR session*)):ti,ab,kw OR leaflet*:ti,ab,kw OR booklet*:ti,ab,kw OR pamphlet*:ti,ab,kw OR poster*:ti,ab,kw OR video*:ti,ab,kw OR ((written OR printed OR oral) near/3 information*):ti,ab,kw OR "academic detailing":ti,ab,kw OR (training next program*):ti,ab,kw OR algorithm*:ti,ab,kw OR (decision next tree*):ti,ab,kw OR (self next care*):ti,ab,kw OR selfcare:ti,ab,kw OR "self-care":ti,ab,kw | **163795** |
| 5 | #3 AND #4 | **1543** |
| 6 | [mh animals] NOT [mh humans] | **7306** |
| 7 | #5 NOT #6 | **1540** |

| Set # | CENTRAL 2020 to 2021 | Results |
| --- | --- | --- |
| 1  Peritoneal Dialysis | [mh "peritoneal dialysis"] OR (peritoneal:ti,ab,kw AND (dialysis:ti,ab,kw OR dialyses:ti,ab,kw)) OR "peritoneal dialysis":ti,ab,kw OR "peritoneal dialyses":ti,ab,kw OR pd:ti,ab,kw OR capd:ti,ab,kw OR ccpd:ti,ab,kw OR apd:ti,ab,kw | **40691** |
| 2  Parkinson  Disease | [mh "parkinson disease"] OR parkinson*:ti,ab,kw | **12012** |
| 3 | #1 NOT #2 | **34843** |
| 4  Patient Education | [mh "patient education as topic"] OR [mh "health education"] OR ((patient* OR health*) near/6 educat*):ti,ab,kw OR "patient education":ti,ab,kw OR "health education":ti,ab,kw OR (patient* near/6 (communicat* OR interacti* OR inform* OR instruct* OR advice* OR advise* OR counsel* OR consel* OR teach* OR train* OR empower*)):ti,ab,kw OR "patient communication":ti,ab,kw OR "patient counseling":ti,ab,kw OR (educat* near/6 (intervention* OR rehabilitation* OR program* OR service* OR group* OR session*)):ti,ab,kw OR leaflet*:ti,ab,kw OR booklet*:ti,ab,kw OR pamphlet*:ti,ab,kw OR poster*:ti,ab,kw OR video*:ti,ab,kw OR ((written OR printed OR oral) near/3 information*):ti,ab,kw OR "academic detailing":ti,ab,kw OR (training next program*):ti,ab,kw OR algorithm*:ti,ab,kw OR (decision next tree*):ti,ab,kw OR (self next care*):ti,ab,kw OR selfcare:ti,ab,kw OR "self-care":ti,ab,kw | **200120** |
| 5 | #3 AND #4 | **1764** |
| 6 | [mh animals] NOT [mh humans] | **33** |
| 7 | #5 NOT #6 | **1764** |
| 8 | Limit with Publication Year from 2020 to 2021, with Cochrane Library publication date from Feb 2020 to Dec 2021, in Trials | **210** |
