# Supplementary material for: Nurse-based educational interventions in patients with peritoneal dialysis: A systematic review and meta-analysis
Source: Int J Nurs Stud Adv. 2022 Sep 24;4:100102. doi: 10.1016/j.ijnsa.2022.100102 (PMC11080474; doi:10.1016/j.ijnsa.2022.100102)
Supplement: Supplementary file 2 [file mmc2.docx]

**Supplementary material 3**

**Fig S3.1.** summary of the authors' judgements on the risk of bias of 10 included randomized controlled trials which were at low, unclear, and high risk of bias as percentages across all included studies.


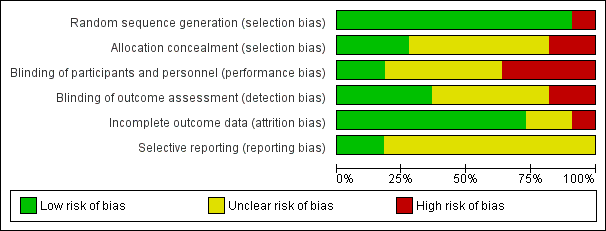


**Fig S3.2.** the authors' judgements on detailed risk of bias of the 10 included randomized controlled trials of nurse-based educational interventions for patients with peritoneal dialysis


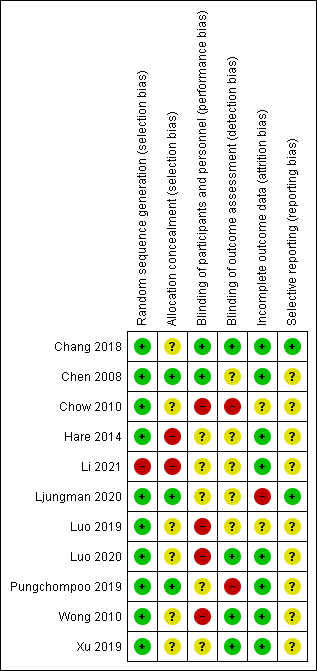


Low risk of Bias

Unclear Risk of Bias

High risk of bias
