# Supplementary material for: Nurse-based educational interventions in patients with peritoneal dialysis: A systematic review and meta-analysis
Source: Int J Nurs Stud Adv. 2022 Sep 24;4:100102. doi: 10.1016/j.ijnsa.2022.100102 (PMC11080474; doi:10.1016/j.ijnsa.2022.100102)
Supplement: Supplementary file 3 [file mmc3.docx]

**Supplementary material 4**

**Figure S4.1.** Forest plot of the risk of peritonitis in patients with PD with subgroup analysis.


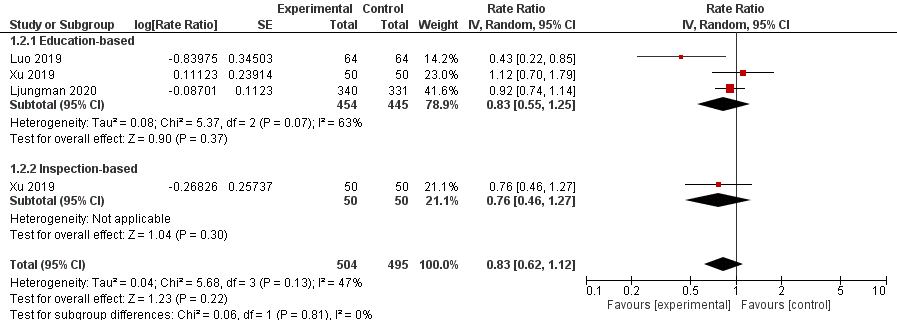


The figure summarizes the risk of peritonitis of PD patients in three eligible studies. The forest plot represents the pooled estimated rate ratio of peritonitis in PD patients (black diamond). The estimated rate ratio for each study was presented with a red diamond), with 95% confidence intervals (95% CI; horizontal black lines). The overall estimated pooled risk of peritonitis was 0.83 (95% CI 0.55– 1.25) for the mainly education-based subgroup analysis. The overall estimated pooled risk of peritonitis was 0.76 (95% CI 0.46– 1.27) for the mainly inspection-based subgroup analysis. The meta-analysis used a random-effects model with the exact method for confidence interval estimation. *I*^2^, test for heterogeneity. df, degrees of freedom; Z, test of overall treatment effect.

**Figure S4.2.** Forest plot of the risk of exit-site infections in patients with PD.


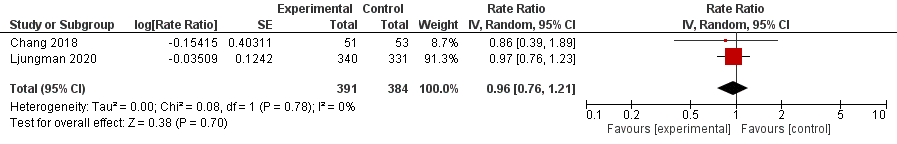

The figure summarizes the risk of exit-site infections of PD patients in two eligible studies. The forest plot represents the pooled estimated rate ratio of exit-site infections in PD patients (black diamond). The estimated rate ratio for each study was presented with a red diamond), with 95% confidence intervals (95% CI; horizontal black lines). The overall estimated pooled risk of exit-site infections was 0.96 (95% CI 0.76– 1.21). The meta-analysis used a random-effects model with the exact method for confidence interval estimation. *I*^2^, test for heterogeneity. df, degrees of freedom; Z, test of overall treatment effect.

**Figure S4.3.** Forest plot of the risk of PD-related infections in patients with PD.


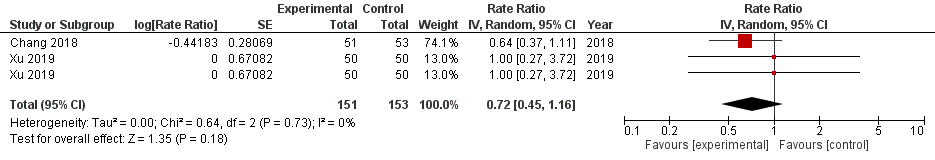

The figure summarizes the risk of PD-related infections of PD patients in two eligible studies (three intervention arms). The forest plot represents the pooled estimated rate ratio of PD-related infections in PD patients (black diamond). The estimated rate ratio for each study was presented with a red diamond), with 95% confidence intervals (95% CI; horizontal black lines). The overall estimated pooled risk of PD-related infections was 0.72 (95% CI 0.45– 1.16). The meta-analysis used a random-effects model with the exact method for confidence interval estimation. *I*^2^, test for heterogeneity. df, degrees of freedom; Z, test of overall treatment effect.

**Figure S4.4.** Forest plot of the risk of death in PD patients

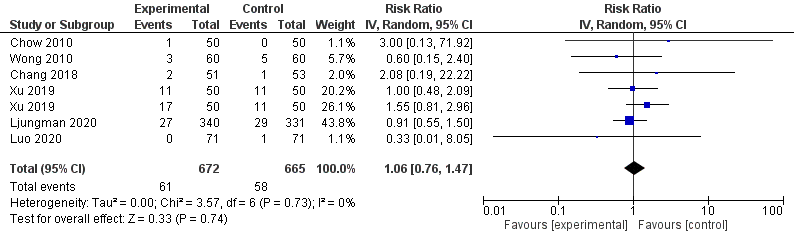


The figure summarizes the risk of death of PD patients in six eligible studies. The forest plot represents the pooled estimated risk ratio of death in PD patients (black diamond). The estimated risk ratio for each study was presented with a blue diamond), with 95% confidence intervals (95% CI; horizontal black lines). The overall estimated pooled risk of death was 1.06 (95% CI 0.76– 1.47). The meta-analysis used a random-effects model with the exact method for confidence interval estimation. *I*^2^, test for heterogeneity. df, degrees of freedom; Z, test of overall treatment effect.
